# Ultrastructural changes associated with the induction of premature chromosome condensation in *Vicia faba* root meristem cells

**DOI:** 10.1007/s00299-014-1637-0

**Published:** 2014-06-05

**Authors:** Dorota Rybaczek

**Affiliations:** Department of Cytophysiology, Institute of Experimental Biology, Faculty of Biology and Environmental Protection, University of Łódź, Pomorska 141/143, 90-236 Lodz, Poland

**Keywords:** Hydroxyurea, Protein kinase and phosphatase inhibitors, Interphase chromatin, PCC-type chromosomes, H3S10 phosphorylation, Transmission electron microscopy

## Abstract

*****Key message***:**

**PCC induction is regulated by several signaling pathways, and all observed effects associated with PCC induction are strongly dependent on the mechanism of action of each PCC inducer used.**

**Abstract:**

Electron microscopic observations of cells with symptoms of premature chromosome condensation (PCC) showed that the interphase chromatin and mitotic chromosomes differed with respect to a chemical compound inducing PCC. Induction of this process under the influence of hydroxyurea and caffeine as well as hydroxyurea and sodium metavanadate led to a slight decrease in interphase chromatin condensation and the formation of chromosomes with a considerably loosened structure in comparison with the control. Incubation in the mixture of hydroxyurea and 2-aminopurine brought about clear chromatin dispersion in interphase and very strong mitotic chromosome condensation. Electron microscopic examinations also revealed the characteristic features of the structural organization of cytoplasm of *Vicia faba* root meristems, which seemed to be dependent on the type of the PCC inducer used. The presence of the following was observed: (i) large plastids filled with starch grains (caffeine), (ii) mitochondria and plastids of electron dense matrix with dilated invaginations of their internal membranes (2-aminopurine), and (iii) large mitochondria of electron clear matrix and plastids containing protein crystals in their interior (sodium metavanadate). Moreover, since caffeine causes either the most effective loosening of chromatin fibrils (within the prematurely condensed chromosomes) or induction of starch formation (in the plastids surrounding the nuclei), this may be a proof that demonstrates the existence of a link between physical accessibility to chromatin and the effectiveness of cellular signaling (e.g., phosphothreonine-connected).

## Introduction

Studies carried out on the organization of S-phase checkpoints elucidated the function of the main elements of the S-M checkpoint, whereby the capability of cells to divide mitotically in response to DNA replication arrest is blocked (Bartek et al. 2004). However, the mechanisms inducing premature chromosome condensation (PCC) through overriding of the S-M checkpoint are relatively poorly recognized (Nghiem et al. 2001; Rybaczek 2011 and ref. therein; Rybaczek and Kowalewicz-Kulbat 2011). Studies carried out by Johnson and Rao (1970) on mammalian cells show that phenomena accompanying PCC—nuclear envelope breakdown, chromatin condensation, and formation of mitotic spindle—are “forced” under the influence of M-phase-promoting factor (MPF) from the cytoplasm of mitotic cells. Our previous work indicated that in the cells blocked during DNA biosynthesis, e.g., by hydroxyurea (HU), the PCC process could be induced by the action of various chemical compounds as follows: (i) protein kinase inhibitors, e.g., caffeine, 2-aminopurine, staurosporine, and wortmannin; (ii) protein phosphatase inhibitors, e.g., sodium metavanadate and calyculin A; (iii) activators of protein kinase C, e.g., 1,2-dioctyl-sn-glycerol; and (iv) DNA topoisomerase II inhibitors, e.g., ICRF-193 (Rybaczek et al. 2002, 2008; Rybaczek and Kowalewicz-Kulbat 2011). The successive phases of prematurely initiated mitosis, and the role of conditioning connected with the specificity of plant cell mitotic divisions, have not yet been fully resolved. Neither the molecular organization of the S-phase checkpoints that block the mitosis initiation nor the mechanism that would make it possible to suppress their restrictive interactions have been well explained. However, the emergence of phospho-H2AX foci at the site of DNA double-stranded breaks (DSBs) in *Raphanus sativus, Vicia faba,* and *Allium porrum* cells induced to enter PCC were found (Rybaczek and Maszewski 2007a, b). Further immunocytochemical observations were performed to evaluate the correlation of H2AX phosphorylation with checkpoint kinase 1 (Chk1) and checkpoint kinase 2 (Chk2) (Rybaczek et al. 2007). The inhibitors used in this study (i.e., caffeine, CF; 2-aminopurine, 2-AP; and sodium metavanadate, Van) block the catalytic functions of various enzymes participating in the transfer of biochemical signals involving DNA biosynthesis (comp. Rybaczek and Kowalewicz-Kulbat 2011). Among these, only CF possesses the capability of inhibiting phosphorylation, which blocks the Cdc25 phosphatase, thereby specifically inducing the PCC process as a result of the activation of cyclin-dependent mitotic kinases. Additional mechanisms must be involved during the PCC induction under the influence of Van or 2-AP, which, despite their inhibitory properties in relation to mitotic kinases, induce the phenomena closely related to the effectiveness of the CDK-dependent apparatus of the phosphorylation of proteins (histones, proteins of division spindle, etc.).

Whether any ultrastructural changes are connected with PCC induction in plants or not has not been studied in detail. The aim of the present study was to determine the influences of HU, CF, 2-AP, and Van on (i) the cell cycle progression, (ii) chromatin condensation (in the aspect of the ultrastructure of interphase cells), and (iii) morphology and ultrastructure of PCC-like chromosomes, in root meristem cells of *V. faba*. The results presented contribute to a better understanding of the initiation of mitosis, the mechanism behind chromatin condensation, and especially premature chromosome condensation.

## Materials and methods

### Plant material and growth conditions

Seeds of *V. faba* (ssp.) *minor* (cv.) Nadwiślański (Center for Seed Production in Sobiejuchy, Poland) were sterilized using sodium hydrochloride (0.3 % v/v) and germinated in Petri dishes on wet Whatman paper at room temperature. Three days after imbibition, dark-grown seedlings with 25-mm-long primary roots were selected for further experiments. During incubation(s) with solutions of HU and during post-treatments, roots were oriented horizontally in a humid chamber and permanently aerated on a rotary water bath shaker (30 rpm) at 23 °C.

### Chemical agents

Hydroxyurea (HU, 2.5 mM), sodium metavanadate (Van, 200 µM), pararosaniline, N-2-hydroxyethylpiperazine-N′-2-ethanesulfonic acid (HEPES), bovine serum albumin (BSA), polyvinylpyrrolidone (PVP-40), propidium iodide (PI), and 4′,6-diamidino-2-phenylindole (DAPI) were purchased from Sigma. Caffeine (CF, 5 mM) was supplied by Merck, Triton X-100 and pectinase from *Aspergillus niger* by Fluka, cellulase Onozuka R-10 from *Trichoderma viride,* and RNase from SERVA. 2-aminopurine (2-AP, 10 mM) and pectolyase Y-23 were obtained from ICN Biomedicals. Other chemicals were obtained from POCH S.A.

### Induction of PCC

Seedlings pretreated for 24 h with 2.5 mM HU were transferred into Petri dishes containing a mixture of (i) 2.5 mM HU and 5 mM CF; (ii) 2.5 mM HU and 10 mM 2-AP; (iii) 2.5 mM HU and 200 µM Van. After 8 h, the root tips were excised and fixed according to the procedure described below (the following paragraph and consecutive paragraphs of “Materials and methods” section).

The control groups in the cytophotometric and microdensitometric analysis consisted of seedlings incubated in water for 24 h (24-h-negative control) or 2.5 mM HU for 24 h (24-h-positive control). The cytophotometry and microdensitometry were the starting point for further immunocytochemical and ultrastructural observation of PCC induction process. As mentioned above, the investigation of morphology and ultrastructure of either PCC-type interphase chromatin or PCC-like chromosomes were performed for successive 8 h of appropriate incubation. On account of this, the control groups in the immunocytochemical and ultrastructural analyses of changes associated with the PCC induction consisted of seedlings incubated in water for 32 h (24 h + 8 h; 32-h-negative control) or 2.5 mM HU for 32 h (24 h + 8 h; 32-h-positive control).

### Cytophotometric and microdensitometric analysis

About 1.5-cm-long apical fragments of primary roots of *V. faba* were fixed in cold Clarke’s mixture (absolute ethanol/glacial acetic acid; 3:1, *v/v*) for 1 h (according to Bruni et al. 1976), washed three times with 96 % ethanol, rehydrated (70–30 % ethanol, distilled water), and submitted to Feulgen staining (according to Rybaczek et al. 2002). For this procedure, the roots were hydrolyzed in 4 M HCl (at room temperature for 2 h) and stained with Schiff’s reagent (pararosaniline). After staining (1 h), root fragments were rinsed three times in SO_2_-water and once in distilled water. About 1.5-mm-long root tips were cut off and squashed in a drop of 45 % acetic acid onto Super-Frost microscope slides (Menzel-Gläser) using the dry ice method. After removing coverslips, the slides were dehydrated, air-dried, and embedded in Canada balsam (Merck, Germany). The nuclear DNA content was evaluated by means of microdensitometry using a Jenamed 2 microscope (Carl Zeiss, Jena, Germany) with the computer-aided Cytophotometer v1.2 (Forel, Lodz, Poland), as described in Winnicki et al. (2013). The extinction of Feulgen-stained cell nuclei was measured at 565 nm and calibrated in arbitrary units, taking the values recorded for half-telophases and prophases from 24-h-negative control plants as reference standard of 2C [26.1 pg; (according to Rybaczek and Maszewski 2007a, and ref. therein)] and 4C DNA levels. To evaluate distribution patterns within the 24-h-negative control and 24-h-positive control, 500 cells were analyzed for each experimental series.

### Flow cytometry analysis

Isolation of cell nuclei was performed according to the method described by Lee and Lin (2005) using (1) MgSO_4_ buffer: 10 mM MgSO_4_ · 7 H_2_O, 50 mM KCl, 5 mM HEPES (pH 8.0); (2) extraction buffer A: MgSO_4_ buffer with 1 % (w/v) polyvinylpyrrolidone (PVP-40), 6.5 mM dithiothreitol, 0.25 % (v/v) Triton X-100; (3) extraction buffer B: MgSO_4_ buffer with 6.5 mM dithiothreitol, 0.25 % (v/v) Triton X-100, 0.2 mg/ml propidium iodide, 1.25 µg/ml RNase (DNase-free). Cell nuclei were filtered using a cotton column. Nuclear DNA content was measured with LSRII flow cytometer (Becton–Dickinson). Experiments for flow cytometry analysis were performed twice with similar results.

### Autoradiographic analysis

The roots of seedlings treated with either 2.5 mM HU for 24 h (24-h-positive control) or incubated in water for 24 h (24-h-negative control) were pulsed for 1 h in 2 ml of 25 µCi cm^−3^ [6-^3^H]thymidine solution (specific activity 40 MBq; Amersham). Then, the seedling roots were rinsed, washed in distilled water for 15 min, and 1 cm segments were fixed for 1 h in cold Clarke’s mixture of absolute ethanol and glacial acetic acid (3:1, *v/v*). After fixation, the root segments were rinsed thoroughly with absolute ethanol, rehydrated, hydrolyzed in 4 M HCl for 2 h, and stained with Shiff’s reagent (pararosaniline) for 1 h (Rybaczek et al. 2002). Root tips (1.5-mm long) were cut off and squashed in a drop of 45 % acetic acid on poly-l-lysine-coated slides (Polysine™; Menzel-Gläser). Slides were covered with Ilford Nuclear Research Emulsion K5D (Polysciences, Inc., Valley Road, Warrington, PA, 18976) and exposed for 20 days, developed, and fixed according to standard methods (Neely and Combs 1976; Heijnen and Geuze 1977). Mitotic and labeling indices were estimated based on the measurements taken from 5,000 cells per sample.

### Cytochemical analysis

Cytochemical detection of starch was performed on the preparations from Feulgen-stained root meristem cells using a standard procedure of staining with Lugol’s solution (I in KI) for 5 min (Polit et al. 2002; Polit 2009). Briefly, 1.5-cm-long apical fragments of primary roots of *V. faba* were fixed in cold Clarke’s mixture (absolute ethanol/glacial acetic acid; 3:1, *v/v*) for 1 h (according to Bruni et al. 1976), washed three times with 96 % ethanol, rehydrated (70–30 % ethanol, distilled water), hydrolyzed in 4 M HCl (2 h), and stained with Schiff’s reagent (pararosaniline). After rinsing in SO_2_-water, 1.5-mm-long apical segments were cut off, washed in distilled water, placed in a drop of 45 % acetic acid, and squashed onto slides. Following freezing with dry ice, coverslips were removed, slides were dehydrated, and embedded in Canada balsam (Merck, Germany). Observations were made using Optiphot-2 microscope (Nikon), and images were recorded by a DXM 1200 CCD camera (Nikon).

### Immunocytochemical analysis

Immunocytochemical detection of phosphothreonine was performed using mouse monoclonal anti-phosphothreonine fluorescein isothiocyanate (FITC)-conjugated antibody (IgG2b isotype, α-T^P^ab-FITC, 1:500), according to Rybaczek et al. (2002). Immunocytochemical detection of phospho-histone H3 (Ser10; H3S10Ph) was performed using anti-phospho-histone H3 (Ser10) antibody (1:400, Cell Signaling), goat anti-rabbit secondary antibody (AlexaFluor488^®^, 1:1,000, Abcam), and the method described by Rybaczek et al. (2007). Briefly, excised 1.5-mm-long apical parts of roots from the control, HU-treated, and PCC-induced seedlings were fixed for 45 min (20 °C) in PBS-buffered 3.7 % paraformaldehyde, washed with PBS, and placed for 45 min (37 °C) in a citric acid-buffered digestion solution (pH 5.0) containing 2.5 % pectinase (Fluka, Germany), 2.5 % cellulase (Onozuka R-10: Serva, Heidelberg, Germany), and 2.5 % pectolyase (ICN, Costa Mesa, CA, USA). After short rinsing with PBS, root tips were squashed onto slides. Air-dried slides were pretreated with PBS-buffered 5 % bovine serum albumin (BSA) at 20 °C for 50 min and incubated for 16 h in a humidified atmosphere (4 °C) in PBS-buffered antibody solution containing 1 % BSA. After washing with PBS (4 times), slides were incubated for 1.5 h (20 °C, in the dark) with secondary antibody in PBS containing 1 % BSA. DNA was visualized with PI (propidium iodide; 0.3 µg mL^−1^) staining. Immunostaining of the cell preparations was recorded by using an Eclipse E-600 fluorescence microscope (Nikon), equipped with U2 filter (UVB light, λ = 340–380 nm) for DAPI (4′, 6-diamidino-2-phenylindole; 0.4 µg mL^−1^), B2 filter (blue light, λ = 465–496 nm) for AlexaFluor488, or G2 filter (green light, λ = 540/25 nm) for PI. All images were recorded by a DXM 1200 CCD camera (Nikon) at exactly the same time of integration.

### Ultrastructural analysis

The apical parts of *V. faba* roots (1.5-mm long) were fixed in 2 % glutaraldehyde in 1 % cacodylate buffer (pH 7.3) for 3 h at 4 °C, postfixed in 1 % osmium tetroxide in the same buffer for 3 h, and dehydrated in an ascending ethanol series. After infiltration with the medium consisting of Epon 812 and Spurr’s resin, ultrathin sections—prepared according to Roland (1978)—were double-stained with uranyl acetate and lead citrate according to Reynolds (1963). The sections were examined and photographed in a JEOL JEM-1010 transmission electron microscope (JEOL, Ltd.).

### Morphometric analysis of ultramicroscopic photographs

Morphometric image analysis was performed to measure the condensed and dispersed chromatin within the whole nucleus area using computer-aided Cytophotometer v1.2 (Forel, Lodz, Poland). Quantitative measurements were based on ultramicroscopic photographs, scanned and inverted (negative). Measurements were taken on at least 100 cell nuclei and averaged (±SD). Quantitative measurements of the degree of loosening of chromatin fibrils were based on ultramicroscopic photographs converted to grayscale and were expressed in arbitrary units as mean pixel value (pv) spanning the range from 0 (dark) to 255 (white), according to the described method (Rybaczek et al. 2007).

### Statistical analysis

Statistical analyses were performed with the STATISTICA 8.0 PL program (StatSoft Inc., Tulsa, Oklahoma). All data were expressed as mean ± SD. Differences between groups were assessed by the nonparametric Mann–Whitney *U* test (for impaired data). Student’s *t* test and Cochran–Cox test were used for data normally distributed. *P* < 0.05 was considered statistically significant (according to Lawnicka et al. 2010).

## Results

### Characteristics of interphase cells in meristems subjected to the action of PCC inducers

From the results presented in Table 1, it follows that in meristems incubated in water for 24 h, one can distinguish three groups of cells: (i) 2C DNA (representing cells in the G1 phase), (ii) 2-4C DNA (representing the cells in the S phase), and (iii) 4C DNA (cells in the G2 phase). The cytophotometric analysis of meristems incubated for 24 h in 2.5 mM HU shows a clear change in the proportions of particular cell classes, among others, the decrease in the G1-phase subpopulation. The results of microdensitometric analyses (Table 2) indicated that the cell nuclei subjected to HU for 24 h (S phase and G2 phase) exhibit different chromatin condensation degree (as compared with the 24-h-negative control). A clear decrease in the condensation level was observed in the nuclei containing 2-4C DNA.

**Table 1:** Percentage of cells at particular phases of interphase and mitosis

Experimental series	Cell cycle phases			
	G1	S	G2	M
Control (%)	28.6	30.5	23.6	17.3
2.5 mM HU, 24 h (%)	0.4	56.8	40.6	2.2

The results of cytophotometric analyses regarding index values calculated on the basis of replication activity analysis (percentage of cells incorporating [6-^3^H]thymidine) as well as the values of mitotic indices

**Table 2:** Average optical density of chromatin (l/pel^2^; arbitrary units) in the interphase chromatin of cells with different contents of DNA; meristems of the control seedlings and those incubated for 24 h in 2.5 mM HU

Experimental series	2C DNA	2-4C DNA	4C DNA
Control	0.14 ± 0.01	0.17 ± 0.01	0.18 ± 0.01
2.5 mM HU, 24 h	–	0.13 ± 0.01	0.2 ± 0.02

**Fig. 1:**
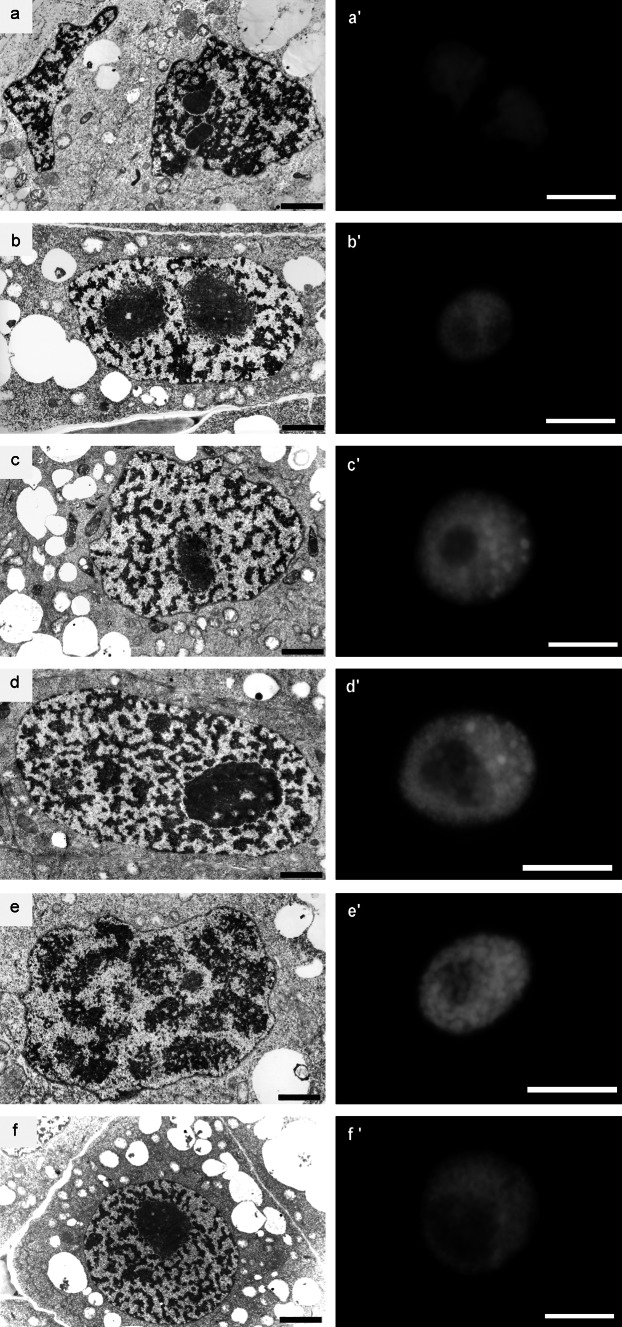
Electron micrographs of *V. faba* cells of control meristems (32-h incubation in water): **a** late telophase; **b** G1 phase; **c** S phase; **d** G2 phase; **e** early prophase. **f** An interphase cell of the *V. faba* root meristem after 32-h hydroxyurea treatment. *Bar* 1 µm. Pictures made from histochemical analyses of DAPI staining (**a′**–**e′** control cells; **f′** hydroxyurea-treated cell). *Bar* 20 µm

Cell nuclei in the meristematic cells of the 24-h-control (type negative) roots were characterized by a considerable difference in shapes, sizes, and ultrastructural chromatin organization. This variability was associated first of all with the course of subsequent cell cycle phases. At the initial stages of interphase, the mitotic chromosomes were gradually decondensed and irregular, and then circular and oval nuclei were formed (Fig. 1b–d). The dispersion of chromatin compact masses was correlated with nucleogenesis and, in cytoplasm, with the fusion of vesicular structures in the central region, where a wall separating daughter cells was observed (Fig. 1a). Establishing the sequence of ultrastructural changes in successive interphase periods proved to be considerably more difficult. Based on serial cross sections of interphase cells (making it possible to perform morphometric estimation of nuclear cell size), one could assume with some confidence that such a sequence has been illustrated, as in the electron microscopic images shown in Fig. 1b–e. Nuclei of small dimensions, representing the G1 phase, were characterized by a relatively low degree of chromatin condensation (Fig. 1a, b). A significant proportion of nucleoplasm volume was occupied by nucleoli with high granular component content which in the peripheral “cortical zone” created fine, isolated aggregates clearly seen on the loose chromatin background. The structural organization of nuclei at later stages of cell cycle (S phase and G2 phase) was characterized by the chromatin concentration processes imparting more and more lattice-like character (Fig. 1c, d). In cells with large nuclei (G2 phase), the space occupied by the condensed chromatin bands constituted about 45 % ±2.24 of the nucleoplasm area cross section, while the nucleoli of these cells were characterized by a strongly condensed structure standing out clearly against the chromatin background (Fig. 1d). The initial stages of mitotic condensation were characterized by the disappearance of areas of electron dense chromatin associated with the internal membrane of nuclear envelope (Fig. 1e). In late-G2 cells (in 2.5 mM HU-treated cells for 32 h; 32-h-positive control), the space occupied by the condensed chromatin bands was about 41 % ±4.26, and the nucleoli were also characterized by strongly condensed structure (Fig. 1f, comp. with Fig. 1d). Additionally, pictures made from histochemical analyses of DAPI staining complete this study well, and authenticate results were obtained on the transmission electron microscopy (TEM; Fig. 1a′–f′).

**Fig. 2:**
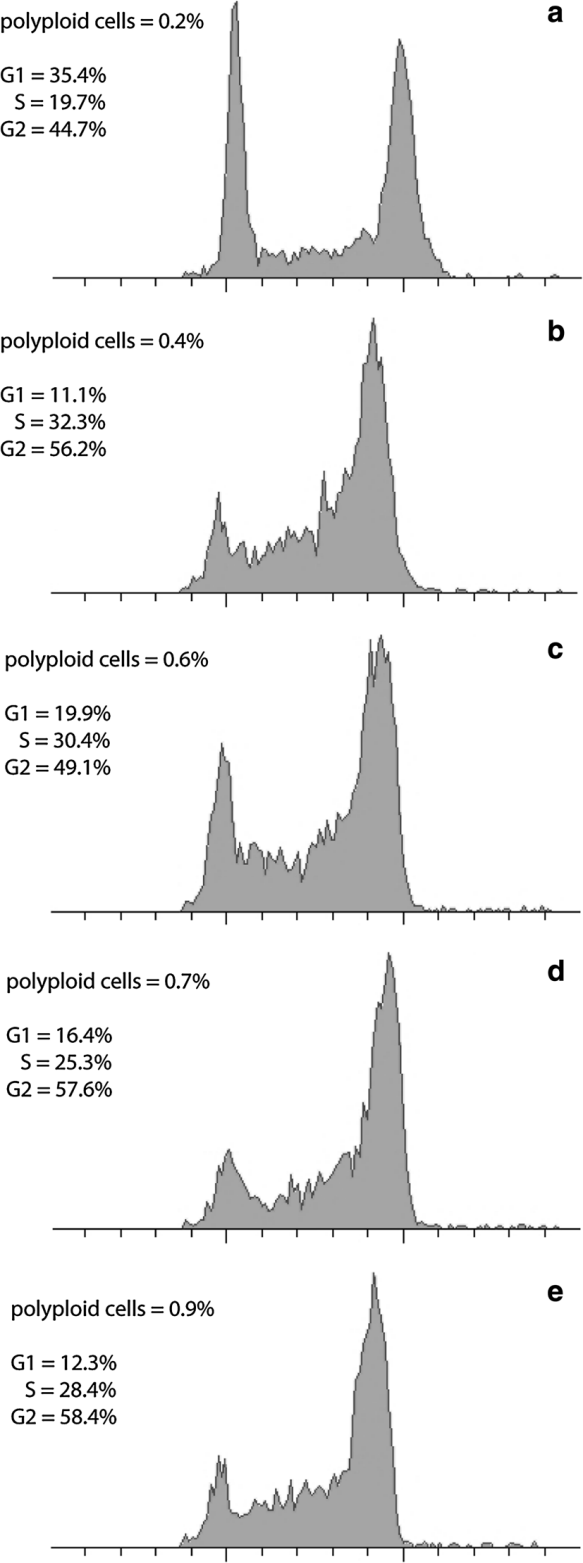
Cell cycle profiles and number of polyploid cells determined by flow cytometry. *V. faba* roots were incubated in **a** water for 32 h, **b** 2.5 mM hydroxyurea for 32 h, **c** 2.5 mM hydroxyurea for 24 h and post-incubated in the mixture of 2.5 mM hydroxyurea and 5 mM caffeine for 8 h, **d** 2.5 mM hydroxyurea for 24 h and post-incubated in the mixture of 2.5 mM hydroxyurea and 10 mM 2-aminopurine for 8 h, **e** 2.5 mM hydroxyurea for 24 h and post-incubated in the mixture of 2.5 mM hydroxyurea and 200 µM sodium metavanadate for 8 h

Flow cytometry analysis revealed that the nuclear DNA content in HU-treated root meristem cells of *V. faba* had shifted toward higher DNA values (Fig. 2b). The same shifts were observed after CF, 2-AP, and Van treatments (Fig. 2c–e). In turn, roots treated with a mixture of HU and CF additionally had increased numbers of cells at early S-phase stages (Fig. 2c). Compared to the negative control (32 h; Fig. 2a), cells incubated either with a mixture of HU/2-AP or HU/Van showed an increase in the number of G2 nuclei (Fig. 2d, e). Notably, in comparison with HU-treated cells (32 h), nearly the same proportion of G1-S-G2 cells was observed in the mixture of HU and Van (Fig. 2b, e). Usual root cells display a high level of polyploidy. In this study, however, in none of the experimental series did we observe more polyploid cells than 1 % of the entire meristem population (Fig. 2a, e). The lowest number of polyploid cells was observed in the control (0.2 %) and the greatest under the influence of HU and Van (0.9 %). Even after the presence of CF, a well-known inhibitor of cytokinesis, a relatively low percentage of polyploid nuclei, was observed (0.6 %). Thus, the correlation between the nuclear size and cell cycle phases was adequate and validated merit (Fig. 1).

**Fig. 3:**
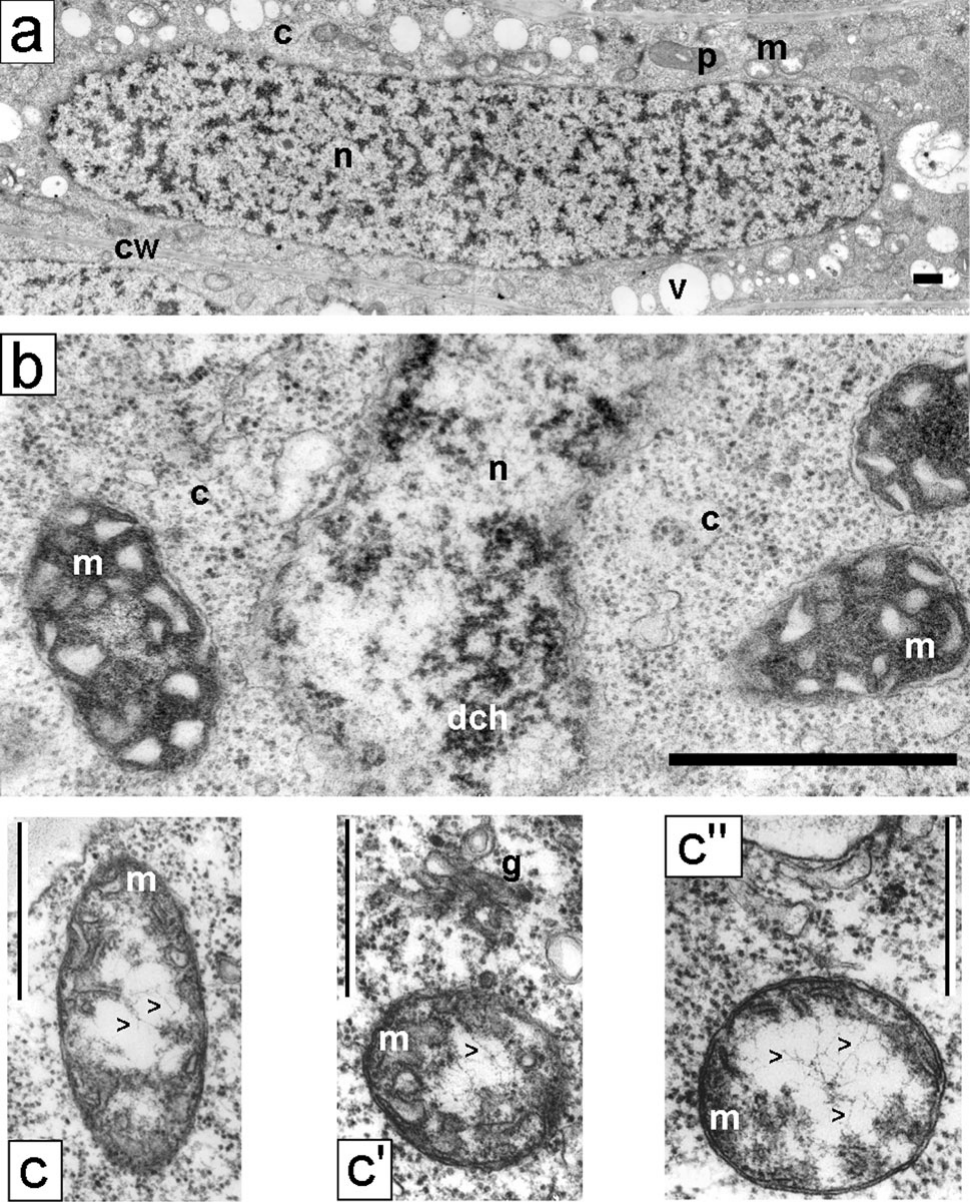
**a** An interphase cell of the *V. faba* root meristem after 24-h replication block and 8-h post-incubation in the mixture of hydroxyurea and caffeine; tetraploid, strongly elongated nucleus with typical chromatin structure, probably resulting from the fusion of two nuclei (effect of caffeine action as cytokinesis inhibitor). **b** A cell fragment after replication block and 8-h post-incubation in the mixture of hydroxyurea and caffeine. The separated area of the nucleus and the cytoplasm with mitochondria with strongly condensed matrix. **c**–**c″** Electron micrographs of mitochondria of control *V. faba* cells; *black arrow* heads indicate DNA fibrils found within the mitochondria; *c* cytoplasm, *cw* cell wall, *dch* dense chromatin, *g* Golgi structure, *m* mitochondrium, *n* nucleus, *p* plastid, *v* vacuole. *Bar* 5 µm

**Fig. 4:**
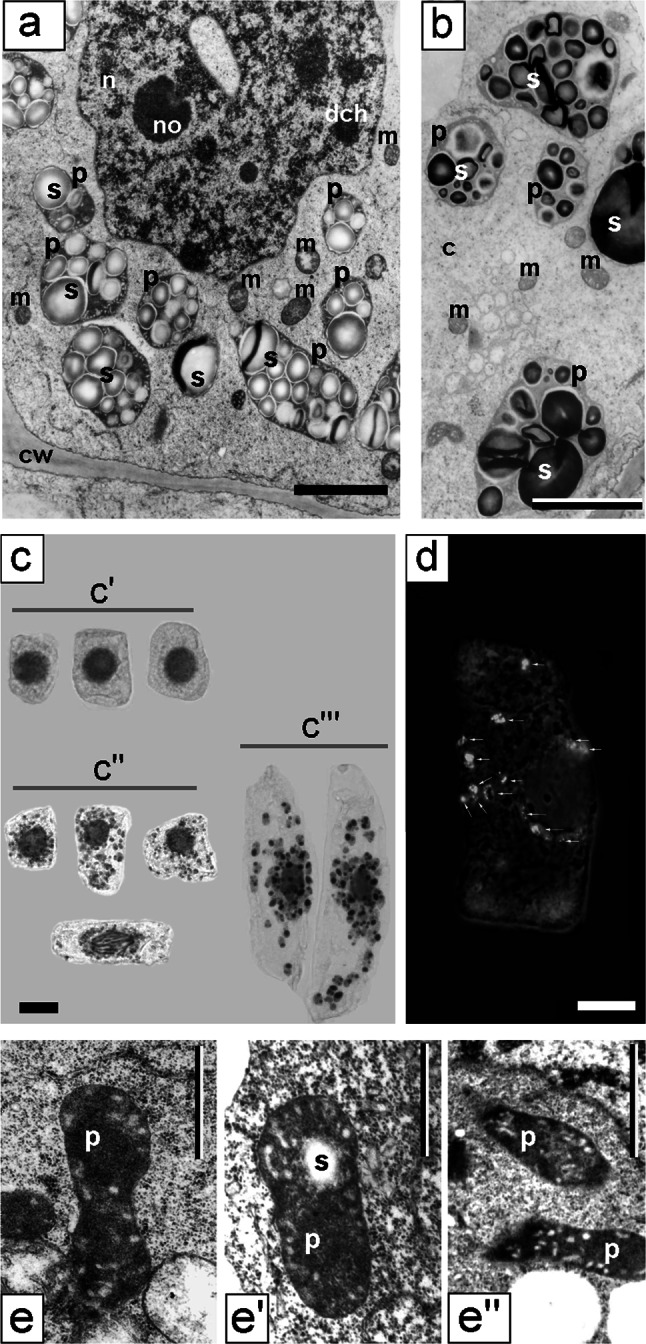
Electron micrographs of meristematic cells from the *V. faba* roots blocked for 24 h in hydroxyurea and post-incubated for 8 h in the hydroxyurea and caffeine mixture. **a, b** Plastids filled with starch grains. Induction of starch formation in the plastids of *V. faba* meristematic cells after 24-h replication block and 8-h post-incubation in hydroxyurea and caffeine mixture. **c** Reaction with Lugol’s solution (*c*′ control cells;* c*″–*c*″′ hydroxyurea/caffeine-treated cells: *c*″ meristematic cells, *c*″′ rhizodermis cells). **d** Fluorescence of starch in plastids (*arrows*) after immunocytochemical reaction with the use of antibodies against phosphothreonine. *Bar* 10 µm. **e**–**e**″ Electron micrographs of plastids of control *V. faba* cells. *c* cytoplasm, *cw* cell wall, *dch* dense chromatin, *m* mitochondrium, *n* nucleus, *no* nucleolus, *p* plastid, *s* starch. *Bar* 2 µm

As opposed to the differentiated morphological images of cell nuclei in the control (type negative) seedling meristems, incubated in water for 32 h, the interphase nuclei in *V. faba* roots subjected to 24-h blocking in 2.5 mM HU and then transferred to HU/CF mixture showed a considerable unification of the structural organization of chromatin (Fig. 3a, b). In almost all nuclei, there were relatively small isolated areas of irregular patches of compact chromatin, irregularly distributed between extensive spaces of loose chromatin. The condensation degree of compact chromatin occupying about 25 % ±1.41 of nucleus cross section area seemed to be lower than that in the cells of 32-h-negative control roots. On the other hand, nucleoli generally showed more condensed structure (Fig. 4a, comp. with Fig. 1d). The ultrastructural image of interphase cell cytoplasm in the roots exposed to CF did show the presence of large plastids filled with numerous starch grains, occupying a considerable portion of cytoplasm (what was the most significant feature of cells of this experimental series; Fig. 4a, b). On the other hand, only slightly more condensed matrix structure was observed in mitochondria, in comparison with either the negative (data not shown) or positive control (Fig. 3b, c′–c″). The mitochondria in meristematic cells of 32-h-negative control root (incubated in water for 32 h) were characterized by a considerable degree of morphological difference. At all interphase stages, their oval forms dominated, showing low degree of matrix condensation and a low number of mitochondrial cristae (Fig. 3c′–c″). Plastids exhibited considerable electron density (Fig. 4a, b, e′–e″). In cells treated with 2.5 mM HU for 32 h (32-h-positive control), the similar types of mitochondria and plastids were observed (data not shown). In CF-treated cells, plastids were clearly separated in specimens stained with Lugol’s solution (Fig. 4c), as well as following immunocytochemical reactions for the presence of phosphothreonine (Fig. 4d).

**Fig. 5:**
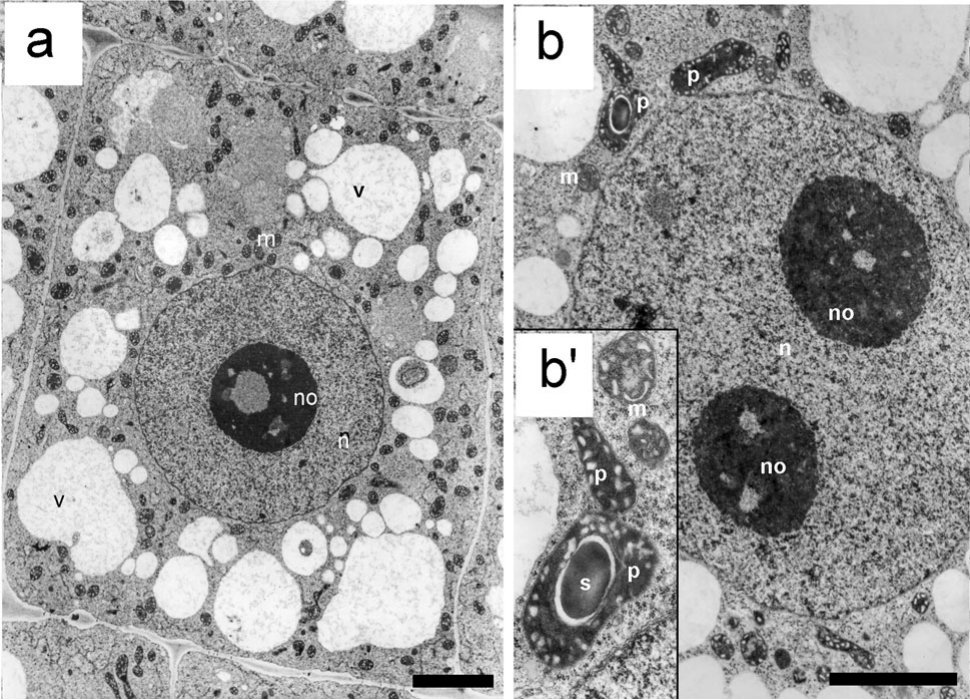
**a**, **b** Electron micrographs of meristematic cells from the *V. faba* roots blocked for 24 h in hydroxyurea and post-incubated for 8 h in the hydroxyurea and 2-aminopurine mixture; **b′** an enlarged fragment of **b**. *m* mitochondria, *n* nucleus, *no* nucleolus, *p* plastid, *s* starch, *v* vacuole. *Bar* 10 µm

Cells subjected to 24-h blocking in 2.5 mM HU and then transferred into HU/2-AP mixture showed a specific kind of unification of the structural organization (Fig. 5a, b). Their nuclei were mostly of regular shapes, and within the whole area, there was strongly decondensed chromatin, owing to which the nuclear envelope was clearly separated. Uniformly dispersed small areas of compact chromatin did not exceed 8 % ±0.95 of the nucleus cross section. Nucleoli were most often condensed, and a clear area was filled with microgranular material contrasted inside them (Fig. 5a, b). The root meristem cells subjected to 2-AP exhibited specific structure of mitochondria and plastids which were filled with electron dense matrix, with light areas of broadened invaginations of internal membranes (Fig. 5b–b′).

**Fig. 6:**
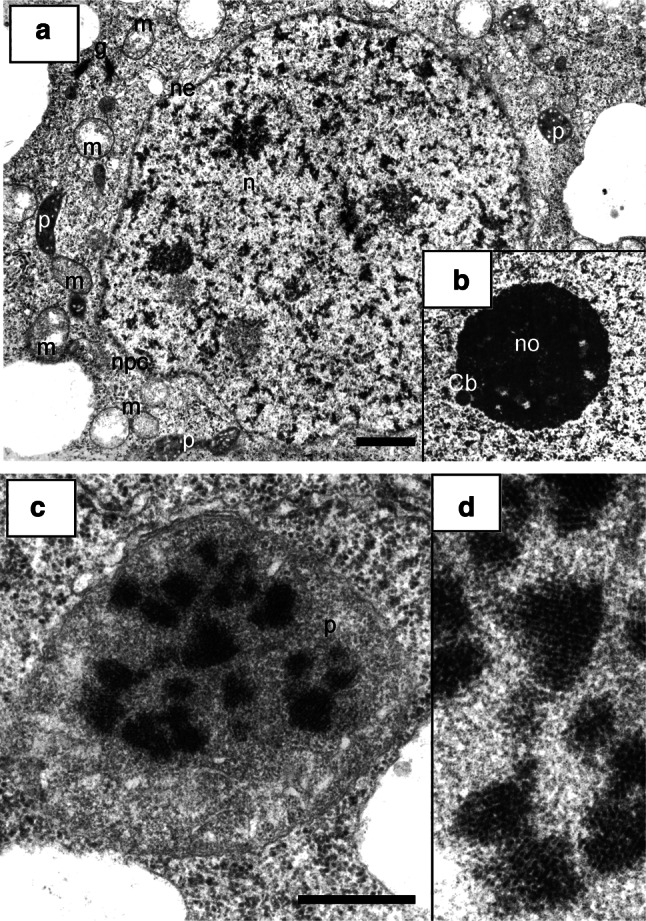
**a**–**d** Electron micrographs of meristematic cells from the *V. faba* roots blocked for 24 h in hydroxyurea and post-incubated for 8 h in the mixture of hydroxyurea and sodium metavanadate. **c**, **d** Protein crystals in the plastid matrix (an enlarged fragment on the right, **d**). *Cb* Cajal body, *g* Golgi structure, *m* mitochondria, *n* nucleus, *ne* nuclear envelope, *no* nucleolus, *npc* nuclear pore complexes, *p* plastid. *Bar* 1 µm

In electron microscopic observations, the ultrastructure of meristematic cells that after the 24-h period of replication block were treated with HU/Van mixture was greatly similar to that of the cells incubated in HU/CF solution (Fig. 6a). However, condensed chromatin occupied smaller cross-sectional areas (about 15 % ±3.31) and its aggregates most often had small dimensions. Regularly shaped nucleoli were strongly condensed (Fig. 6b). Mitochondria possessing relatively large dimensions were characterized by electron-transparent matrices (Fig. 6a), while inside plastids, proteins were often visible (Fig. 6c, d).

### Ultrastructure of PCC-like chromosomes

**Fig. 7:**
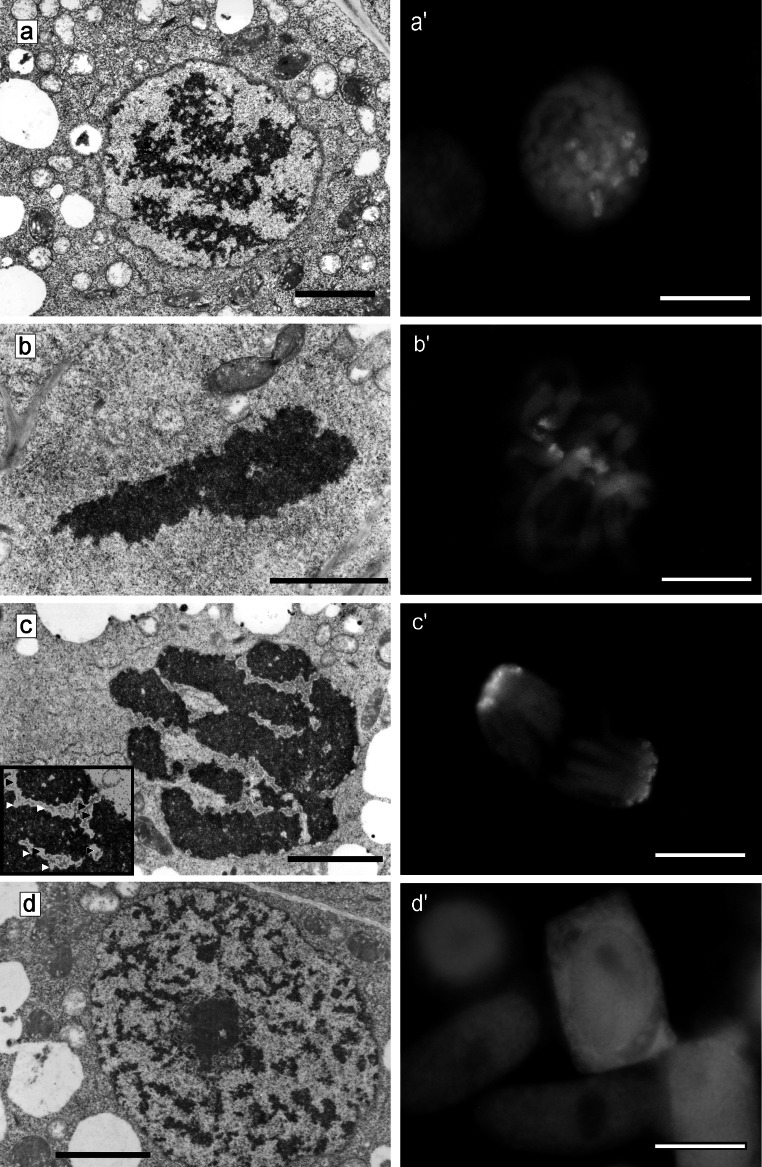
Electron micrographs of *V. faba* cells of control meristems (32-h incubation in water): **a** a cell in prophase; **b** metaphase chromosome; **c** a group of chromosomes in anaphase; the beginning of nuclear envelope formation. Light areolas on the boundary of chromatin–cytoplasm are indicated by *arrows*. **d** An interphase cell of the *V. faba* root meristem after 32-h hydroxyurea treatment. *Bar* 5 µm. Immunofluorescence of phosphorylated histone H3 (Ser10; H3S10Ph) in control root meristem cells of *V. faba* (*green signals*) and PI-stained nuclei: **a**′ prophase; **b**′ metaphase; **c**′ anaphase; as well as **d**′ in the cell nuclei from hydroxyurea-treated roots (for 32 h). *Bar* 20 µm (color figure online)

Figure 7a–c illustrates consecutive mitosis stages (prophase–metaphase–anaphase) in the meristematic cells of control roots (32-h incubation in water). The structure of prophase nuclei, separated from cytoplasm with a clear envelope, was non-uniform, and cross sections of chromosomes were clearly visible against the background of surrounding nucleoplasm in the form of lattice-like structures exhibiting a high degree of electron density (Fig. 7a). The final, uniform, and compact structure of chromosomes was formed in the metaphase (Fig. 7b). A similar degree of chromosome condensation was maintained in the anaphase. In this case, however, the character of chromatin–cytoplasm contact changed in the polar region (light areolas were formed; Fig. 7, arrows), which seemed to be a symptom of initial stages of nuclear envelope reconstruction (Fig. 7c). Additionally, Fig. 7a′–c′ illustrates H3S10 histone phosphorylation at consecutive mitosis stages (from prophase, Fig. 7a′—through metaphase, Fig. 7b′—to anaphase, Fig. 7c′). It also shows no phosphorylation during G2/M cell cycle transition in the roots treated with 2.5 mM HU for 32 h (positive control, Fig. 7d–d′).

**Fig. 8:**
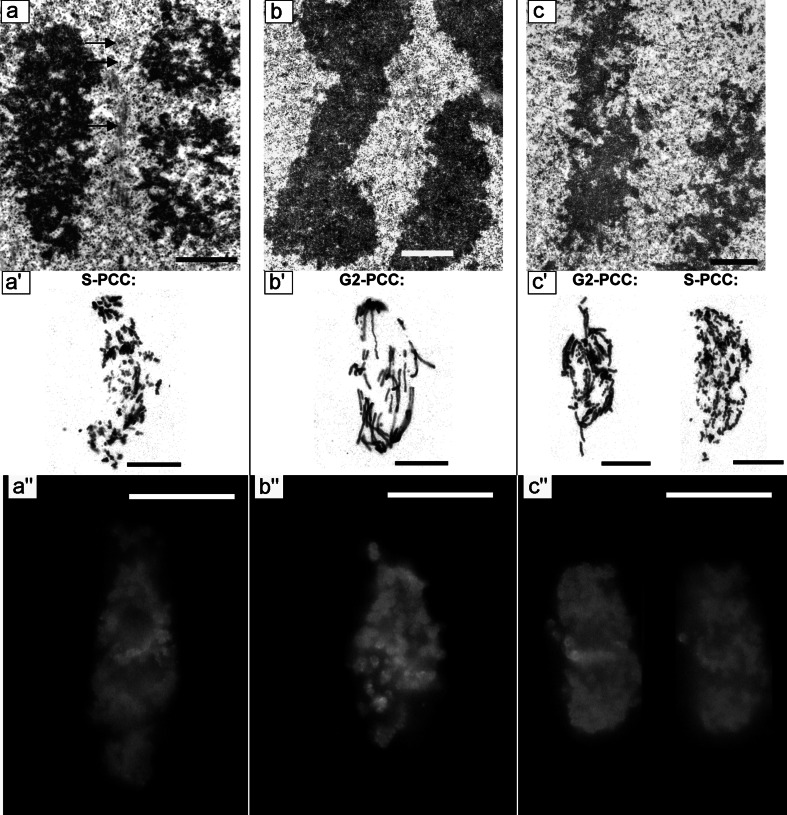
PCC induced by caffeine–anaphase chromosomes. Microtubules visible between chromosomes (indicated by *arrows*, **a**); **a**′ S phase induced PCC after Feulgen staining; **a″** immunofluorescence of phosphorylated histone H3 (Ser10; H3S10Ph). **b** PCC induced by 2-aminopurine—anaphase chromosomes; **b′** G2 phase induced PCC after Feulgen staining; **b″** immunofluorescence of phosphorylated histone H3 (Ser10; H3S10Ph). **c** PCC induced by sodium metavanadate—anaphase chromosomes; **c**′ G2 phase induced PCC after Feulgen staining (*left*) and S phase induced PCC (*right*) after Feulgen staining; **c**″ Immunofluorescence of phosphorylated histone H3 (Ser10; H3S10Ph). *Bars* 1 µm (**a**, **b** and **c**), 10 µm (**a**′, **b**′, **c**′), 20 µm (**a″**, **b″**, **c″**)

At none of the PCC-type mitosis stages induced by the action of CF, did the level of chromosome condensation reach the level of chromatin packing observed in control cell chromosomes (incubated in water for 32 h). Short chromosome fragments in the phenotype-C cells (S-phase-induced PCC leading to chromosome pulverization; S-PCC) showed irregular outlines and numerous clearing ups (Fig. 8a–a′). Microtubules were visible between the chromosomes (Fig. 8a; arrows). The 2-AP-induced PCC exclusively concerned the mitotic figures qualified to phenotype B (G2-phase-induced PCC leading to the losses of large fragments of chromosomes caused a relatively small number of breakpoints: <20 per cell nucleus; G2-PCC) (Fig. 8b′). This was also reflected at the ultrastructural level as the chromosome morphology of PCC-like B-type phenotype (G2-PCC) was just slightly changed in comparison with the control (incubated in water for 32 h) (Fig. 8b; comp. with Fig. 7c). Chromosomes in the cells induced to PCC by the action of Van showed particular ultrastructural differences (both phenotype B and phenotype C of prematurely induced mitoses were observed; G2-PCC and S-PCC) (Fig. 8c–c′). An excellent marker in additional phenotyping PCC induced from various subperiods of S phase (from early to late) was H3S10Ph (Fig. 8a″–c″).

**Fig. 9:**
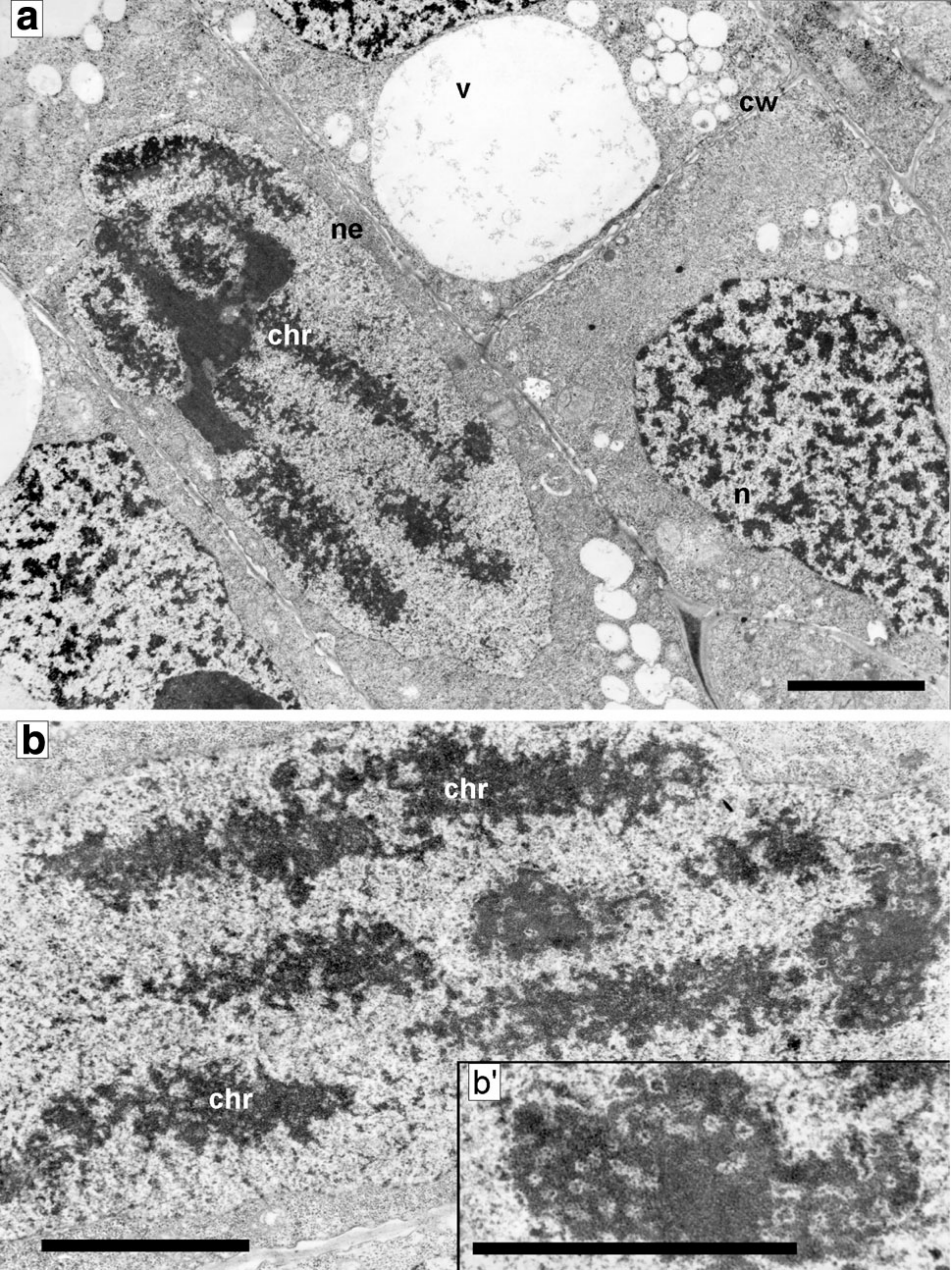
** a**,** b** Electron micrographs of meristematic cells from the *V. faba* roots blocked for 24 h in hydroxyurea and post-incubated for 8 h in the hydroxyurea and sodium metavanadate mixture. Prophase cells—the differentiation of chromosome condensation degree. **b′** an enlarged fragment of **b**. *chr* chromosome, *cw* cell wall, *n* nucleus, *ne* nuclear envelope, *v* vacuole. *Bar* 10 µm

Interestingly, in prophase of Van-treated cells, one could observe arms of condensing chromosomes similar to those in the root cells exposed to CF as well as chromatin, in the form of a compact dense mass of exceptionally low electron permeability (Fig. 9a, b). Sometimes, clearings on chromosome borders could be observed, while in the centers small areas of higher density could be seen (Fig. 9b′).

**Fig. 10:**
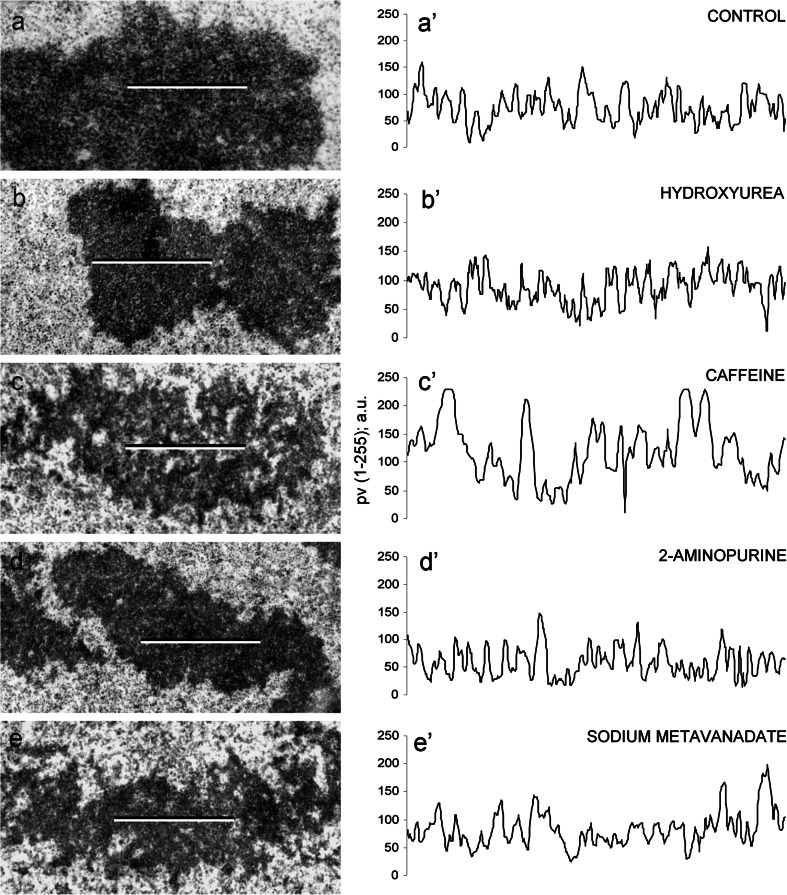
**a–e** Profiles of electron density of chromosomes scanned along the lines marked in the left panel. **a′**–**e′** Quantitative measurements were expressed in arbitrary units as mean pixel value (pv) spanning the range from 0 (*dark*) to 255 (*white*). “Zero” level of the electron density was determined inside a vacuole

**Fig. 11:**
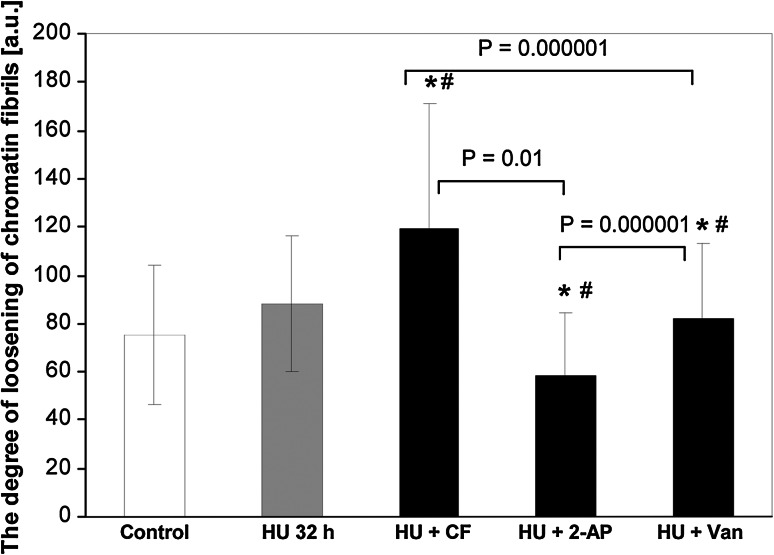
Degree of loosening of chromatin fibrils [arbitrary units, a.u]. *Columns*, mean from the three independent experiments; *bars*, SD. * *P* < 0.005, compared with control (32-h incubation in water), *#P* < 0.005, compared with hydroxyurea (32 h)

The electron density profiles of metaphase chromosome sections selected from the control cells incubated in water for 32 h (a–a′), HU treatment for 32 h (b–b′), HU/CF-induced PCC (c–c′), HU/2-AP-induced PCC (d–d′), and HU/Van-induced PCC (e–e′) are shown in Fig. 10 to indicate the degree of chromosome condensation following different drug treatments. Chromosomes formed after HU and HU/2-AP treatments (Fig. 10b–b′ and d–d′) were even more condensed than normal (32-h-negative control) chromosomes (Fig. 10a–a′). On the other hand, HU/Van-induced chromosomes were more pulverized (Fig. 10e–e′), while electron density within HU/CF-generated chromosomes was the most pronounced (Fig. 10c–c′). As shown in Fig. 11, a correlation was found between the 32-h-negative control and the following experimental series: HU → HU + CF (Cochran–Cox test), HU → HU + 2-AP (Student’s *t* test), HU → HU + Van (Cochran–Cox test), as well as between the cells treated with HU for 32 h (32-h-positive control) and the following experimental series: HU → HU + CF (Cochran–Cox test), HU → HU + 2-AP (Student’s *t* test), HU → HU + Van (Cochran–Cox test). All the other correlations (showed in Fig. 11) were significant (*P* < 0.01) for all experimental series reported herein (Mann–Whitney *U* test). It is noteworthy that statistical analysis strongly emphasizes that CF, as the most effective PCC inducer, also caused the greatest loosening of chromatin fibrils in phenotype-C cells (S-PCC; *P* < 0.0001).

## Discussion

The incorporation of *V. faba* root meristems into the state of replication block (HU treatment), being the starting point of tests with the use of PCC inducers (CF, 2-AP, Van treatments), undoubtedly did not arrest the cell cycle in two closely defined stages of the S phase and G2 phase, but instead drastically slowed down the course of cell cycle progression, engaging the molecular “intervention” mechanisms of the checkpoints in both phases (Rybaczek et al. 2008, comp. Hartsuiker et al. 2001). However, Winnicki et al. (2013) revealed G1/S arrest in root meristems of *V. faba* treated for 24 h with 2.5 mM HU. Cytophotometric quantitative measurements of nuclear DNA after the 24-h replication block revealed two classes of cells: (i) those with 2-4C DNA content characterized by a low coefficient of chromatin condensation, and (ii) those with near 4C DNA content showing considerably greater condensation (comp. with Rybaczek et al. 2008). The difference in the DNA condensation coefficients calculated for both these subpopulations was great enough to explain not only the “effect” resulting from the measurement method (the increase in the measured extinction connected with the higher DNA content was not compensated with the parametric increase in the nucleus profile) but, it would seem, the origin of real differences in the structural organization of cell nuclei. This conclusion seemed to be supported by the level of chromatin condensation in the 24-h-negative control meristem cells, where the differences between the DNA condensation coefficients in the nuclei from phase G1 (2C) and phase G2 (4C) were considerably smaller.

**Fig. 12:**
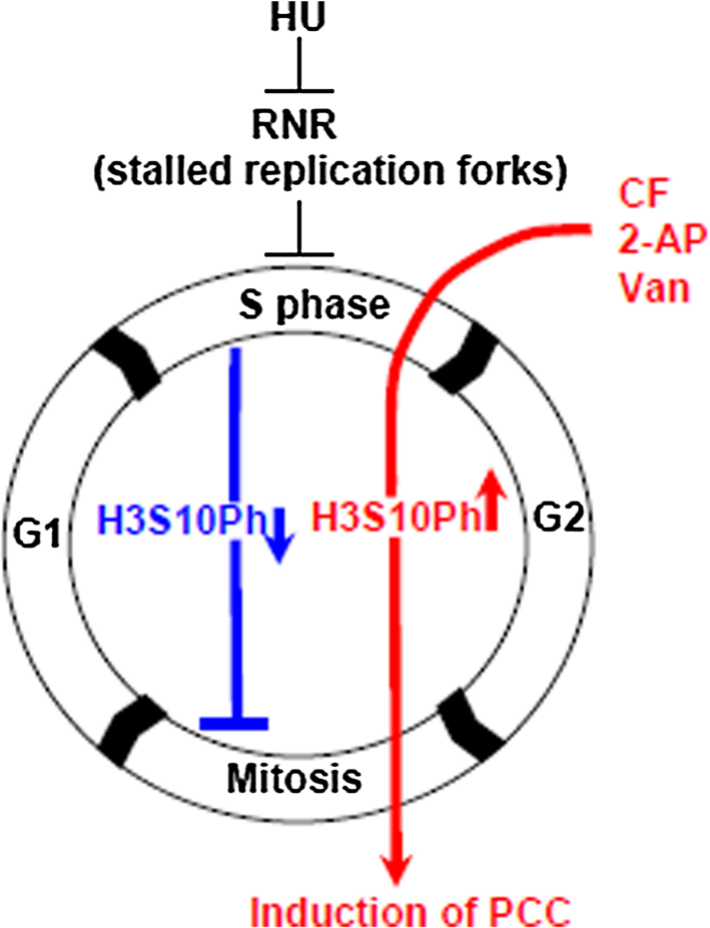
A model for hydroxyurea (HU)-induced S-phase inhibition as well as the induction of premature chromosome condensation (PCC) via caffeine (CF), 2-aminopurine (2-AP), and sodium metavanadate (Van) action, together with the indications of the degree of H3S10 phosphorylation. HU, an inhibitor of ribonucleotide reductase (RNR), inhibits DNA replication-connected fork progression. The lack of RNR activity leads to the inhibition of the S phase and consequently to the inhibition of entry into mitosis. Under the influence of HU, serine 10 of H3 histones remains mainly unphosphorylated (*blue* pathway). CF, 2-AP, and Van induce PCC, and its attendant strong phosphorylation of H3S10 (the hallmark of mitotic condensation; *red* pathway) (color figure online)

Significant differences appeared in the ultrastructural image of interphase cells depending on the PCC inducer used: CF, 2-AP, or Van. The heterogeneity of reactions resulting from the induction of PCC with various chemical agents, measured in real time, reflects differences occurring within the cell “macro-population” present in the root meristem. Additionally, the appearance of PCC inducers such as CF, 2-AP, or Van (acts in combination with HU) mobilized specific biochemical signals connected with the mechanism of action of every single PCC inducer used (Fig. 12; comp. Rybaczek and Kowalewicz-Kulbat 2011). With the transition from the block in the S phase to the former state, visible PCC symptoms (8 h; comp. Rybaczek et al. 2008) lasted considerably longer than the G2 → PCC transition (4 h; comp. Rybaczek et al. 2008). However, neither the former nor the latter transitions were synchronic while both were prolonged in time, manifesting their “internal heterogeneity.” It is a typical phenomenon occurring in apical zones of roots in many plant species (Steeves and Sussex 1991). Physiological responses to external stimuli in certain cells depend not only on the force causing a given reaction (e.g., the local concentration of inducer) but also on (i) the effect of previous interactions, (ii) the cell position within the meristem, (iii) local influences of adjacent cells, (iv) the cell cycle phase (or subperiod of the S phase) in which the cell is under the influence of the inhibitor (HU), and also (v) the cell’s metabolic state. Presumably, the above factors also provide some of the reasons for the low effectiveness of the PCC induction in *V. faba m*eristems (comp. with Nghiem et al. 2001; Rybaczek and Maszewski 2007a; Rybaczek et al. 2008; Rybaczek and Kowalewicz-Kulbat 2011). On the other hand, during PCC induction after CF, 2-AP, and Van treatments, phosphorylation at S10 of H3 histones was observed (Figs. 8, 12) clearly demonstrating that general rules of normal cell cycle progression were well preserved here, indicating that CF, 2-AP, and Van induce PCC and all its attendant H3S10Ph (comp. Rybaczek 2011). The posttranslation modification of H3 histone consisting of phosphorylation at Ser10 (H3S10Ph) constitutes a preliminary signal of mitotic-type chromatin condensation and is tightly correlated with prophase initiation [in plant cells, the phosphorylation of Ser10 histone H3 takes place in prophase chromosomes which are already condensed (Kaszás and Cande 2000) and does not extend beyond the pericentromeric region (Houben et al. 1999; Manzanero et al. 2000)]. Phosphorylation of N-terminal fragments of molecules of histone H3 (by Aurora kinases [Demidov et al. 2009]) provides conditions for the assembly of other proteins—those directly involved in chromatin structural transformations (e.g., condensin complex, [Wei et al. 1999]). The exception among plant cells are interphase-mitotic (IM) cells showing gradual changes in chromatin condensation obtained following continuous 72-h treatment of *Allium cepa* seedlings with 0.75 mM HU. These kinds of IM cells showed the immunolabeling of H3S10Ph extended in time and scale (not confined to the periods ranging from early prophase to ana–telophase, but also in the G2 phase and in late telophase). Additionally, in the late telophase, pericentromeric immunofluorescence disappeared and new strongly labeled phosphorylation sites emerged at telomeres (Żabka et al. 2012). In animal cells, H3S10Ph histones are often already observed in the early stages of the G2 phase, in regions occupied by pericentromeric heterochromatin (Zhang et al. 2005), and spread gradually toward telomeres, covering (during the metaphase) the entire area of chromosome arms. Loss of histone H3 Ser10 phosphorylation occurs during the ana–telophase transition and is associated with the start of telophase decondensation of chromosomes (Hendzel et al. 1997). In lateral root tips of *V. faba* mitotic-type hyperphosphorylation of histone H3 at Ser10 was observed after microcystin-LR (MCY-LR) exposure (either during long-term exposure or short-term MCY-LR treatment). In both these cases, however, H3S10Ph was not detectable in interphase cells (Beyer et al. 2012). In mentioning research on H3S10Ph, one cannot omit the results obtained by Schroeder-Reiter et al. (2003) who presented magnificent images obtained after high-resolution detection of H3S10Ph in mitotic barley chromosomes using scanning electron microscopic technique. It is a novel application of indirect immunogold labeling with Nanogold promising for high-resolution ultrastructural studies of chromosomes. Backscattered electron (BSE) images of signals were superimposed by authors (Schroeder-Reiter et al. 2003) onto gray-scale topographic secondary electron (SE) images. Not surprisingly, in metaphases, signal was clearly detectable in the pericentromeric region bordering the stretched, parallel matrix fibrils of the centromere.

One of the objectives of the electron microscopic examinations was, in this context, to ascertain whether the interphase cells (the greater portion of the meristematic population) show any symptoms of ultrastructural differences in the initiation time of PCC. Opinions concerning the action of CF as PCC inductor are constantly changing, and the mechanism of releasing a cell from the S-M dependency by this alkaloid has not been completely explained but some of the data describe CF as an inhibitor of ATM/ATR kinases (Blasina et al. 1999; Sarkaria et al. 1999; Moser et al. 2000; Zhou et al. 2000; Winnicki and Maszewski 2012). Paweletz et al. (1995) used a mixture of HU and CF in the research of immunoelectron microscopy (immuno-EM) on the organization of chromatin in the centromere–kinetochore complexes of Chinese hamster ovary (CHO) cells. 2-AP is a synthetic purine derivative, an analogue of adenine, showing antagonistic effects in relation to protein kinases [it blocks the activation processes of p34^cdc2^ (Andreassen and Margolis 1994), c-*fos,* and c-*myc* proto-oncogenes as well as the phosphorylation of pRb; (Wu and Gilbert 1997 and ref. therein)]. The blockage of epithelial mouse cells in the S phase under the influence of Van is a consequence of the activation of those biochemical systems, whose central factors are p53 and p21 proteins. The DNA damage caused by the action of Van can provide a pathway of signal transduction implicating the family of protein kinases related to this pathway, leading to the increase in the content of p53 protein and its phosphorylation. The target protein in relation to p53 is p21 inhibitor (Waf1/Cip1). The induction of p21 can inhibit the proliferation of cells, and this effect may be the result, among other things, of the inhibiting effect of numerous complexes of protein kinases and cyclins. The p21 protein inhibits the activity of cyclin A/Cdk2 complex, whose function consists of phosphorylating the E2F transcription factor to reduce its capacity for combining with chromatin. Seen in light of such clues, one can assume that the action of Van results more from the inhibition of protein phosphatase activity than from the interactions connected with the cell replication apparatus (Zhang et al. 2002).

In comparison with the lattice-like structure of chromatin in the 32-h-negative control root meristems dominating almost the whole interphase, the morphology of cell nuclei in the meristems subjected to the PCC inducing action of CF was changed only slightly. The basic difference concerned the chromatin condensation; it was clearly of smaller extent, causing the compact chromatin bands to undergo fragmentation and assume an “islet” character. The chromatin dispersion processes manifest themselves even more clearly in the meristem cells treated with the HU/Van mixture, while a particularly strong decondensation was manifested under the influence of the HU/2-AP mixture. The reduction in the areas of compact chromatin in the electron microscopic specimens of meristematic *V. faba* root cells incubated with the three inhibitors used can result from their specific reaction to closely defined biochemical pathways (Fig. 12, comp. with Rybaczek and Kowalewicz-Kulbat 2011). Thus, CF-induced effects might reflect the antagonism between compounds of methylxanthine character and calcium ions; this antagonism is, among others, the cause of CF-triggered inhibition of cytokinesis in plant cells (Samuels and Staehelin 1996). Calcium plays the role of a signal transmitter participating in the regulation of many metabolic processes associated with the action of a phytochrome, the functioning of stomata, proliferation of hairy root tip cells, as well as pollen tubes and algal rhizoids (cited by Sanders et al. 1999). This ion also plays a direct role in the activation of key transcription control mechanisms as well as in the functioning of enzymes connected with the control of the cell cycle and chromatin condensation (Dudits et al. 1998; Patel et al. 1999).

In the series, in which PCC was induced by 5 mM CF, one could observe the accumulation of phosphothreonine-positive plastids filled with starch grains. Also in the studies carried out by Polit (2009), attempting to elucidate the causes of diversified sensitivity of cells to the inhibitors of protein kinases and phosphatases used during G1-S and G2-M transitions, it has been shown that the induced phosphothreonine-positive areas are plastids accumulating starch grains (after the treatment of *V. faba* cells with 6-DMAP but not with OA). Polit (2009) has confirmed the results obtained by ultrastructural examinations that revealed the around-nucleus location of starch-rich plastids in control cells, and has confirmed their lack in the carbohydrate-starved cells. The carbohydrate-starved cells were strongly vacuolizated, while the plastids containing no starch grains were pushed into peripheral regions of the cell. After 3 h of regeneration in sucrose, the irregular plastids, often with elongated shapes, returned close by the nucleus, showing then the presence of newly synthesized fine starch gains that just after 12 h of regeneration completely filled the interior of plastids. Ultimately, it has been concluded that the around-nucleus location of plastids can be connected with the still not settled plastid-to-nucleus signaling in meristem cells (Polit 2009). Given the above results, the observed phosphorylation of threonine (in HU/CF-treated cells) and its location in plastids may have contributed to the mobilization of sugars in the cell, necessary for CF-induced transitions in the cell life cycle. On one hand, it may be associated with circumventing the function of checkpoints and PCC-like induction of mitosis, and on the other hand with the cell’s attempts to achieve complete replication and post-replication repair in G2. According to the results obtained by Polit and Ciereszko (2009), the metabolic activity of *V. faba* plastids was additionally connected with sugar transformations, as was indicated by the localization of hexokinase (HK) and fructokinase (FK) closely to or even inside plastids. As that work showed, both enzymes (HK and FK) are responsible for sugar metabolism in the cell as well as signaling (especially those biochemical pathways that are responsible for the activation of replication and/or mitosis). Threonine residues undergo phosphorylation through the action of a threonine kinase. Functional genomic-connected analysis demonstrates that Ser/Thr kinases (and phosphatases) are not only involved in the regulation of cell cycle progression, but primarily in DNA damage-related signaling (Reinhardt and Yaffe 2013).

The dispersion of interphase chromatin induced by the influence of Van might be connected with the disappearance of kinase p34^*cdc2*^/H1 activity and dephosphorylation of H1 and H3 histones (Ajiro et al. 1996; comp. with Fig. 9c–c″); the plant equivalent of phosphatase Cdc25 seemed to be the mediator of these reactions (Landrieu et al. 2004a, b). The same role, resulting from the direct inhibitory influence on the activity of cyclin-dependent kinases responsible for the interphase form of chromatin, might be also played by 2-AP. Probably, in this case, the decondensated state of chromatin was possibly not the only symptom of DNA matrix activation. One of the clearest changes induced in the meristematic cells of *V. faba* under the influence of HU/2-AP (observable also after 8-h incubations in the HU/CF and HU/Van mixtures) was the condensation of mitochondria. In this case, disappearance of polymorphism affected the whole population of these organelles indicating the change in their functional state. The electron dense image of mitochondria structure observed in various cell systems in situ—as opposed to their decondensed “orthodox” form—is assumed to reflect their high activity in the process of oxidative phosphorylation (Kwiatkowska et al. 1980; Kwiatkowska 1981). However, one cannot exclude the possibility that the condensation of mitochondria matrix was correlated with chromatin decondensation, with both of these processes resulting from a specific modification of the general balance of phosphorylation and dephosphorylation of cell proteins. Thus, it seems that besides the general relations between the activity of protein kinases (mainly CDK), phosphorylation level of histones, and chromatin condensation degree, recognized first of all in the context of mitotic processes, it is now difficult to refer to analogous examples of ultrastructural modifications of cell nuclei in other representatives of higher Eukaryota. However, for many reasons, mainly those related to methodology, the problem of regulation mechanisms of interphase chromatin structure in the cell cycle undoubtedly belongs among the most difficult issues facing contemporary cell biology (Iborra and Cook 2002).

In this study, it has been shown that during the PCC induction with Van (200 µM), protein crystals are accumulated in the interior of plastids. The crystalline inclusions of proteinaceous character were often observed in plant cells, as located either within cell organelles (such as plastids, mitochondria, endoplasmic reticulum, microbodies, or cell nucleus) or free in the cytoplasm (Briarty et al. 1968; Bosabalidis and Papadopoulos 1983, and ref. therein). It seems reasonable to conclude that the appearance of protein crystals in various cell compartments may be associated with the cell’s response to stress factors. However, this hypothesis remains to be proven. Perhaps, an analysis of recombinant proteins in plant cells (Stöger et al. 2001; Arcalis et al. 2004) may help clarify some of the still-unsolved questions, such as protein structure, function, packaging, trafficking, and importance.

It was previously shown that CF and/or 2-AP and/or Van are able to induce PCC in G1, S, or G2 phase blocked cells in plants (Sen and Ghosh 1998; Rybaczek et al. 2002, 2008), animals (Terzoudi et al. 2010), and humans (Nghiem et al. 2001; Rybaczek and Kowalewicz-Kulbat 2011). However, apart from the description of PCC symptoms and some attempts to understand the mechanism(s) of PCC induction, the influence of either initiation of PCC or the course of PCC on the remaining compartments of the studied cells (apart from the nucleus) has not been characterized. This study demonstrates CF-induced PCC is associated with a simultaneous mobilization of sugars in a cell. This result is an indirect confirmation of research by Reinhardt and Yaffe (2013) on the phosphorylation of threonine residues, performed by threonine kinases in response to the detection of DNA damage (known as DNA damage-related phosphorylation). So far, no paper has reported links between phosphothreonine-connected signaling and the generation of starch in plastids in the context of PCC induction (even in research using CF, the classical PCC inducer). The results of our study suggest that in approaching PCC induction, it is essential to widen the focus from the nucleus to the entire cell. This is why detailed comparative interphase analyses (made under electron microscopy but referred also to histochemical and immunocytochemical analyses) are a promising tool in detecting new dependencies in intracellular signaling pathways that are responsible for maintaining genome stability.

## Conclusion

The presented results have led to the following conclusions: (1) the initiation of premature mitoses, and the distinct characteristics of particular experimental series variations of both index values, seems to point to considerable physiological heterogeneity of the reactions of particular cell subpopulations to the inhibitors used (comp. Rybaczek et al. 2008). This might result from the heterogeneity of meristem checkpoint organization and consequently from the differentiated effectiveness of the factors able to overcome their functions (supervision of proper replication course, post-replication DNA repair, or the control of its structure integrity); (2) the course of PCC induction is a complex matter this being the consequence of many overlapping effects, some of which result from the difference in metabolic states, cell dimensions, and their location within meristem; (3) ultrastructural, immunocytochemical, and histochemical changes observed here can be seen as a product of the interrelation (interplay) between elements of the checkpoint-connected biochemical pathways (activated after the action of the applied PCC inducers) and those factors that typically underlie the chromatin condensation/decondensation processes (e.g., H3 histone S10 phosphorylation).
